# Isotopically Enriched Layers for Quantum Computers Formed by ^28^Si Implantation and Layer Exchange

**DOI:** 10.1021/acsami.3c01112

**Published:** 2023-04-19

**Authors:** Ella Schneider, Jonathan England

**Affiliations:** Surrey Ion Beam Centre, Advanced Technology Institute, University of Surrey, Guildford GU2 7XH, United Kingdom

**Keywords:** ion implantation, isotopically pure layers, metal-induced layer exchange, quantum computing, semiconductor

## Abstract

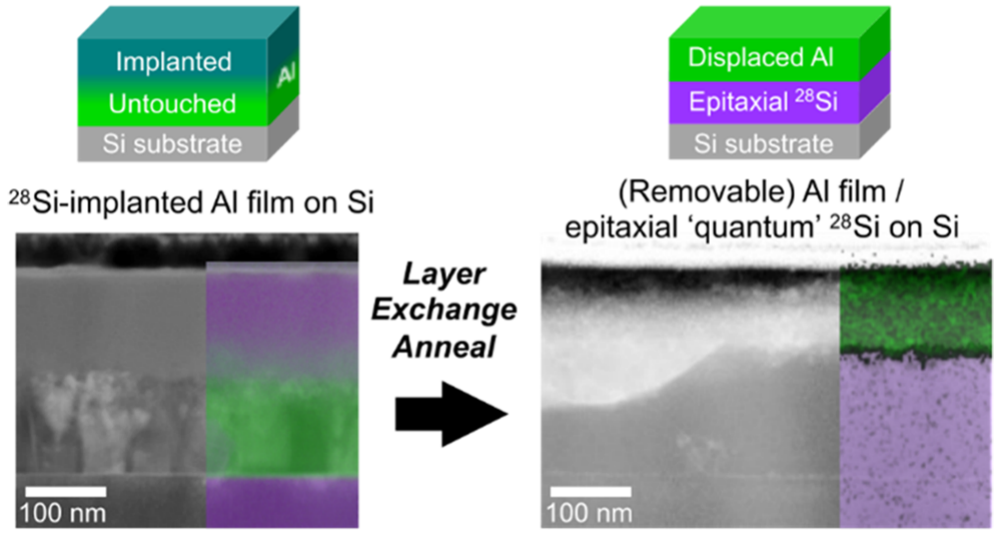

^28^Si enrichment is crucial for production of group IV semiconductor-based quantum computers. Cryogenically cooled, monocrystalline ^28^Si is a spin-free, vacuum-like environment where qubits are protected from sources of decoherence that cause loss of quantum information. Currently, ^28^Si enrichment techniques rely on deposition of centrifuged SiF_4_ gas, the source of which is not widely available, or bespoke ion implantation methods. Previously, conventional ion implantation into ^natural^Si substrates has produced heavily oxidized ^28^Si layers. Here we report on a novel enrichment process involving ion implantation of ^28^Si into Al films deposited on native-oxide free Si substrates followed by layer exchange crystallization. We measured continuous, oxygen-free epitaxial ^28^Si enriched to 99.7%. Increases in isotopic enrichment are possible, and improvements in crystal quality, aluminum content, and thickness uniformity are required before the process can be considered viable. TRIDYN models, used to model 30 keV ^28^Si implants into Al to understand the observed post-implant layers and to investigate the implanted layer exchange process window over different energy and vacuum conditions, showed that the implanted layer exchange process is insensitive to implantation energy and would increase in efficiency with oxygen concentrations in the implanter end-station by reducing sputtering. Required implant fluences are an order of magnitude lower than those required for enrichment by direct ^28^Si implants into Si and can be chosen to control the final thickness of the enriched layer. We show that implanted layer exchange could potentially produce quantum grade ^28^Si using conventional semiconductor foundry equipment within production-worthy time scales.

## Introduction

This paper reports the formation of a continuous layer of enriched ^28^Si for quantum technologies using an aluminum layer exchange process combined with ion implantation. A readily available source of “quantum-grade ^28^Si” with properties suitable to support quantum devices is essential now for research and in the future for mass production of quantum computers. Our process that uses conventional semiconductor foundry equipment for surface cleaning, deposition, implantation, and annealing opens up the possibility for volume manufacture of enriched Si substrates and layers in standard CMOS foundries.

Spin qubits isolated from the environment in “quantum-grade” silicon are attractive quantum computing devices due to their long coherence times, scalability, and potential compatibility with industrial CMOS manufacturing.^1−4^ Qubit spins states, associated with electrons or holes confined in quantum dots^2,5^ or around donor^1,4^ or acceptor^6^ atoms or associated with nuclei,^1,4^ must be isolated from the environment so that quantum computational operations can be performed. As ^28^Si atoms themselves have no spin, an isotopically pure layer of ^28^Si can be cryogenically cooled to act as a “solid-state vacuum” in which spin states can be isolated. Naturally occurring silicon (^natural^Si, composed of 92.2% ^28^Si) contains 4.7% ^29^Si which, possessing nuclear spin, can decohere qubit spin states. Many quantum devices are made in quantum grade Si that is enriched to contain 800 ppm ^29^Si.^5,7−12^ The remaining 3.1% of atoms in ^natural^Si are ^30^Si which, although spinless, should be eliminated because differences in isotopically dependent bond lengths cause strain variability within qubit environments, widening the cross-chip qubit operational parameters such as NMR/ESR transition frequencies.^13,14^

Other causes of spin decoherence must also be made as small as possible. Common contaminant atoms such as C, N, and O possess spin and so must be reduced to levels <10 ppm.^15^ Bulk lattice defects can introduce sources of decoherence from their spin, charge, or strain and impose the need for single-crystal material with low defectivity. Additional defects at layer interfaces introduce two further requirements. For qubits that rely on being placed near an interface,^4^ the growth of high quality dielectrics onto Si implies the enriched layer should have a surface roughness <0.2 nm (root mean square).^11^ Other qubit strategies^1,4^ that place the spin particle remotely from such a noise source require sufficiently thick (>50 nm for ^31^P donors in ^28^Si) layers to allow this.

Early quantum device researchers sourced ^28^Si from excess stocks prepared for the Avogadro Project^15^ which determined Avogadro’s constant by manufacturing an isotopically pure 5 kg bulk ^28^Si sphere using centrifuge-based enrichment of SiF_4_ converted to pure, single crystals via CVD deposition and float zone purification.^16−19^ Contamination levels in this purified material were measured to contain 10 ppm ^29^Si, 0.01 ppm O, 0.1 ppm C, 0.0001 ppm B, and 0.001 ppm P.^16^ More recently, successful quantum devices have been made in quantum grade Si that was made by conventional epitaxy of centrifuge enriched SiF_4_ onto Si wafers.^11,15,20^ This material contained 800 ppm ^29^Si with elemental contamination levels undetectable by SIMS and no crystal defects observed in cross-sectional TEM lamellae.

Another enrichment approach has been to use electromagnetic isotope separation equipment which can operate in three energy regimes.^21,22^ The US National Institute of Standards and Technology has shown that low energy (<3 keV) ^28^Si^+^ could be directly deposited onto the surface of ^natural^Si substrates to produce enriched ^28^Si layers with <1 ppm residual ^29^Si using a purpose built “hyperthermal” ion beam system.^23−26^ Ultrahigh-vacuum levels were required to avoid oxidation of the deposited layer, but even then O (and C) concentrations were >1 × 10^19^ cm^–3^.^23^ Si beam currents (and hence process throughput) were limited to 620 nA^25^ at the low beam energies used. To improve throughput, the University of Surrey explored increasing beam energies up to 20 keV where the use of conventional high current ion implanters could be possible.^21^ Si beam transport could be significantly improved (potentially into the ∼20 mA regime) and surface layer oxidation avoided by implanting the ^28^Si into the body of a ^natural^Si substrate.^21^ However, the high Si self-sputtering rate (>1 ^28^Si atoms/ion) in this energy regime limited the achievable enrichment level,^21^ and isobaric ^14^N_2_^+^ and ^12^C^16^O^+^ (and even ^56^Fe^2+^) mass contamination from the implanter was found to be present. The University of Melbourne^22^ further increased the beam energy to 45 keV where the advantages of increased beam current and silicon enrichment remote from the surface to avoid oxidation continued, and significantly, the Si self-sputtering yield dropped below unity. The enrichment level, no longer dictated by self-sputtering, was now governed by the dilution of the ^29^Si and ^30^Si present in the region of the ^natural^Si being enriched. This higher energy approach required relatively high fluences (2.6 × 10^18^ cm^–2^) to reach an enrichment of 250 ppm ^29^Si. Negative ions were used to avoid introducing isobaric molecular contamination in the ^28^Si beam. The 100 nm thick enriched Si regions with 250 ppm of residual ^29^Si were created after a ^28^Si fluence of 2.63 × 10^18^ cm^–2^ with further optimization possible.^22^

**Scheme 1:**
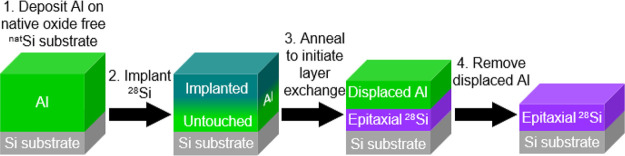
Implanted Layer Exchange (ILE) Process

This paper reports on implanted layer exchange (ILE)—a silicon enrichment process using implantation and aluminum layer exchange (Scheme 1) that reduces required implant fluences by an order of magnitude and avoids oxidation and some contamination issues associated with conventional implantation.

Conventional, deposition-only, aluminum layer exchange has been used to form large-grained polycrystalline Si layers at low temperatures (∼500 °C) in the fabrication of low-cost solar cells on glass.^27,28^ The deposition-only process entailed depositing polycrystalline Al on a glass substrate followed by deposition of amorphous Si onto the Al. An anneal at 500 °C for ∼1 h dissolved Si into the Al which diffused along the Al grain boundaries. During the anneal, the diffusing Si could nucleate, usually heterogeneously on favorable crystal grain boundaries sites, causing Si grains to form, grow, and then Ostwald ripen until a continuous, large-grained poly-Si layer was formed on the glass. Majni and Ottaviani^29^ briefly reported that epitaxial layers could be formed when deposition layer exchange was performed on a crystalline Si wafer as opposed to a glass panel. In both processes, the layer exchange process was driven by the higher Gibbs free energy of the deposited amorphous Si compared to the post-exchange crystalline Si.^14^ Our implant-based layer exchange enrichment process leverages the conventional layer exchange approach but replaces the step of depositing Si onto Al with a ^28^Si implant into the Al layer. The layer exchange anneal causes diffusion of the implanted ^28^Si through the Al layer and allow the ^28^Si to grow epitaxially onto the crystalline Si substrate wafer. A preliminary investigation of ^28^Si implanted into a 500 nm Al film followed by an anneal at 400 °C for 3 h had only formed discrete Si grains in the aluminum as the implanted Si was not able to diffuse through the Al film and crystallize on the substrate as it was too thick.^30^

The aim of this study was to investigate if implantation of Si into Al using a conventional implanter followed by a subsequent layer exchange process could epitaxially grow continuous enriched layers sufficiently thick (>50 nm) and with contamination levels (particularly oxygen and aluminum) and crystal lattice defectivity low enough to be considered quantum grade. For the process to be ultimately viable ILE should be shown to promise credible throughput, have a wide process window, and be insensitive to vacuum conditions.

## Results and Discussion

ILE was investigated at the University of Surrey using a conventional ion implanter to implant 30 keV ^28^Si to a fluence of 6.6 × 10^17^cm^–2^ into Al layers of 100, 150, and 250 nm followed by two 30 s anneals of 500 °C. Samples were imaged after the implant and anneal using top-down optical microscopy, SEM, and cross-sectional TEM. Their elemental and isotopic contents were measured using STEM-EDX, and ToF-SIMS is used to characterize the layers after the implantation and exchange crystallization steps of the ILE process. A more complete description of the fabrication and metrology methods is given in the Experimental Section.

TRIDYN^25^ modeling was used to help interpret the implant results (growth and depth of Si penetration and increase of film thickness) and SIMS measurements (effect of mixing by sputtering beam). We also used TRIDYN to investigate the process window in terms of implantation energy, vacuum level, and fluence to predict the potential and compare the process window of the ILE process to that of direct implantation into Si.^22,21^ The parameters used in the TRIDYN modeling are described in the Computational Modeling section.

### Post-Implantation Measurements

**Figure 1:**
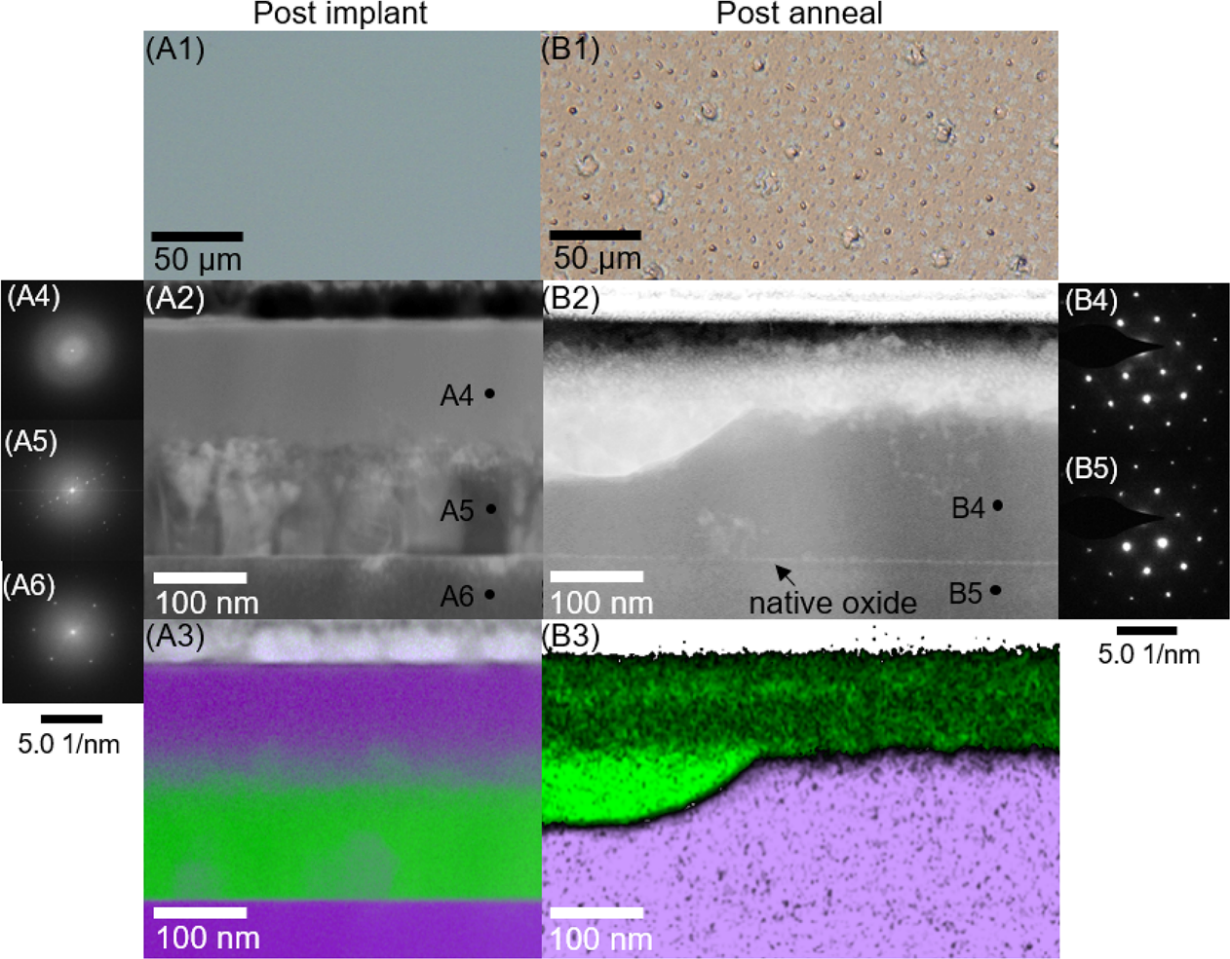
Top-down optical imaging (A1, B1) and cross-sectional DF STEM images (A2, B2) of an initially 150 nm thick Al layer (A) post-implantation and (B) after a layer exchange anneal of two cycles of 30 s at 500 °C. False color EDX maps of Si (purple) and Al (green) reveal a post-implant Si rich layer above an untouched Al and Si substrate (A3) and that during the anneal the Si had exchanged position with Al and grown on the Si substrate (B3). High-resolution TEM fast Fourier transform diffraction patterns show that the implanted region is amorphous (A4), the untouched Al is polycrystalline (A5), and the substrate is single crystalline (A6). The similarity of the nanobeam diffraction patterns measured in the exchanged layer (B4) and Si substrate (B5) indicates the ^28^Si growth was epitaxial. The locations of the diffraction measurements are shown in (A2, B2).

**Figure 2:**
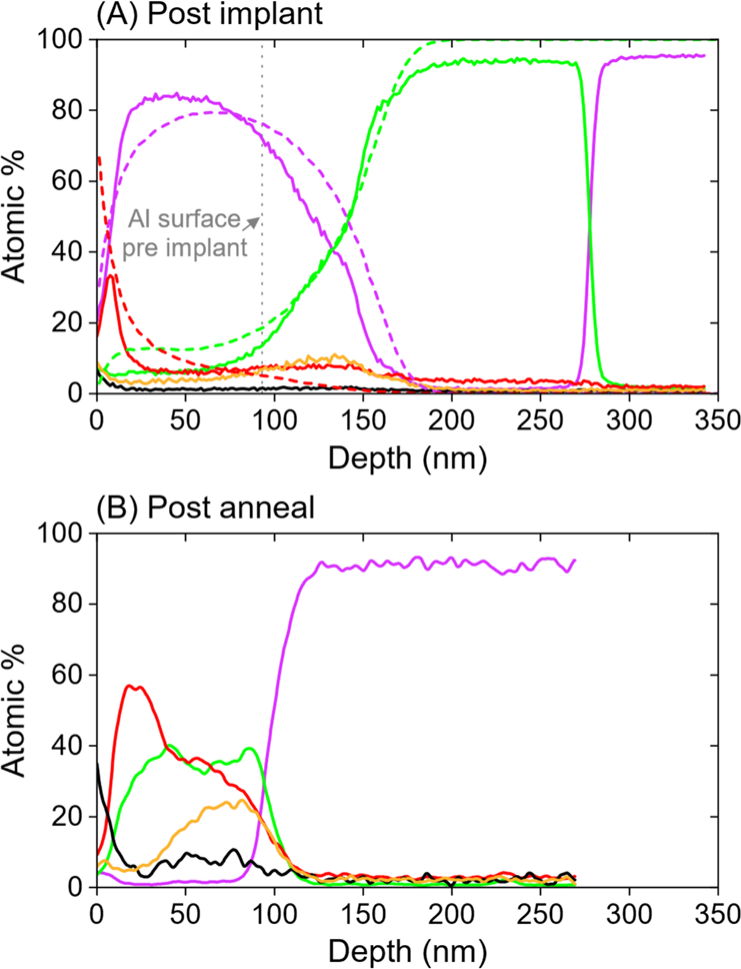
Results of implant and layer exchange of an initially 150 nm thick Al layer. (A) STEM-EDX depth profile of the 150 nm Al layer implanted with ^28^Si/30 keV/6.6 × 10^17^ cm^–2^ (extracted from Figure 1A3). Profiles are shown for Si (purple), Al (green), O (red), N (orange), and C (black). The dashed lines show a TRIDYN model of the ^28^Si implant. The best fitting model included a flux of zero energy O equal to the 30 keV Si flux. (B) STEM-EDX line profile of 150 nm Al implanted with ^28^Si/30 keV/6.6 × 10^17^ cm^–2^ and annealed with two cycles of 30 s 500 °C (extracted from Figure 1B3).

Implantation of Si into the deposited Al film produced a uniform result across the sample as observed in top-down optical images (Figure 1A1). The post-implant cross-sectional images and profiles of Figures 1 and 2 show that an ∼150 nm thick, Si rich (up to 84% Si) amorphous surface layer was produced above an untouched part of the original Al film.

TRIDYN modeling of the implant in Figure 2A showed that the 30 keV ^28^Si^+^ ions penetrated the deposited Al film up to ∼100 nm from the original Al layer surface, and the TEM image of Figure 1A2 shows that the crystal structure of the Al grains untouched by the implanted Si ions and substrate interface remained intact. This was important for success of the subsequent layer exchange process. If the Al layer is equal to or less than the Si ion range, the substrate interface can be amorphized after which subsequent layer exchange fails. Section II of the Supporting Information shows the analysis of an implanted 100 nm thick Al film in which ILE had failed for this reason. TRIDYN modeling of the implant (Figure 2A) showed that around 100 nm of material was deposited during the implant, and EDX measurements show that N and O contamination was incorporated due to implantation directly of accelerated mass 28 isobars N_2_^+^ and CO^+^ and recoil implantation of residual vacuum species absorbed onto the surface of the sample throughout the implantation. We attribute the oxygen measured in the untouched Al (180 to 280 nm) as being due to oxidation of the lamella.

Careful inspection of the false color image in Figure 1A6 shows the presence of Si crystallites in the 150 nm Al film beyond the range of the implant. This indicated that Si diffusion and crystallization could proceed even during the implant and will need to be controlled for the best ILE outcome.

### Post-Layer Exchange Anneal Measurements

The implanted Si-rich amorphous surface layer was subsequently converted into a crystalline layer on the substrate by a layer exchange anneal. The top-down optical microscopy image in Figure 1B1 shows that the uniformity of the post-implanted layer changed during the layer exchange anneal; a uniform background now contained many circular (and some irregular) features of varying size. The TEM cross sections of Figure 1B2,B3 and EDX measurements of Figure 2B were taken in the planar background regions and show that the background represented where successful layer exchange with epitaxial growth onto the substrate had occurred. The images show that an exchanged Si layer was now located immediately above the substrate interface with Al displaced to the surface. The Si substrate interface was identified by a visible thin, sharp interface line which may have been caused by residual native oxide or other surface contamination prior to Al deposition. Nanobeam diffraction patterns (Figure 1B4,B5) confirmed epitaxial single-crystal growth of the exchanged Si layer onto the substrate. Some areas of contrast in the images of the exchanged Si layers indicated the presence of crystal defects in the exchanged layer

Complementary top-down SEM and optical images (for another anneal condition of 500 °C for 1 h) shown in the Supporting Information confirmed that the features projected out of the background layer. Cross-sectional TEM imaging across the dark circular features (such as that shown in Figure S2) showed the features to be regions in which epitaxial growth had been confounded with nucleation and growth of large Si or Al grains proceeding beneath the implanted layer.

### Gettering of O, C, and N Contamination

The EDX line scan of Figure 2B shows that Al, C, N, and O concentrations in the exchanged ^28^Si above the substrate (region 120–260 nm) were below the 1% detection limit of STEM-EDX. The location of the isobaric C, N, and O contamination in annealed samples (Figure 2B) suggests that gettering to implantation damage occurred during heating. TRIM^31^ calculations also showed that implanted ions leave behind interstitial Al defects that peak in concentration at ∼25 nm from the sample surface. The net effect, accounting from the deposition of material during the implant, is that interstitial defects are distributed up to ∼125 nm below the final surface. Comparing the STEM-EDX postanneal profile (Figure 2B) to the as-implanted (Figure 2A) shows that the O, N, and C contamination has diffused ∼70 nm toward the location of interstitial defects after annealing for 1 min. The large O peak at the surface is attributable to surface oxidation of the exchanged Al. This suggests that isobaric contamination was gettered to implant damage during layer exchange annealing and was excluded from the enriched ^28^Si layer.

### Isotopic Enrichment

**Figure 3:**
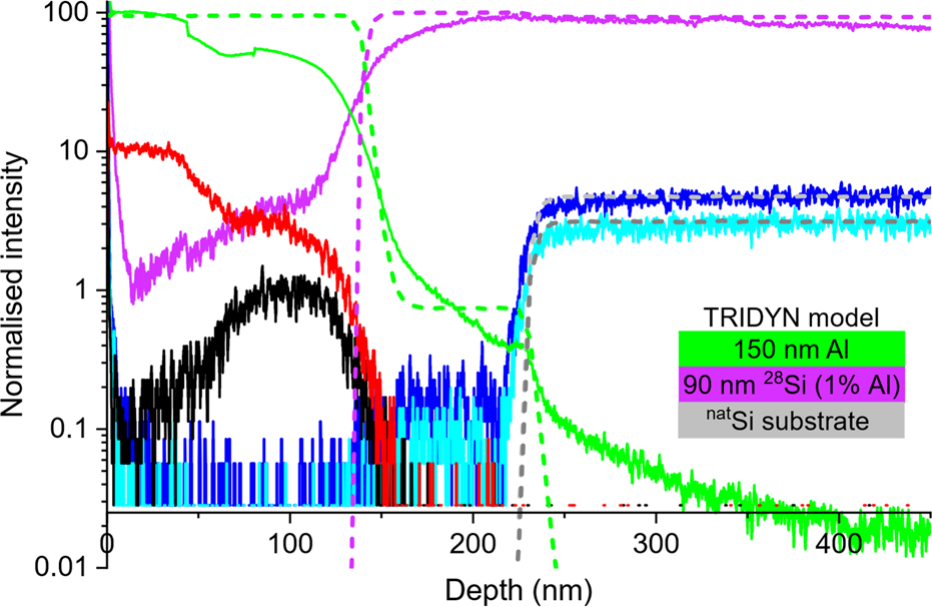
ToF-SIMS measurement (solid lines) of 150 nm Al implanted with ^28^Si/30 keV/6.6 × 10^17^ cm^–2^ annealed annealed with two cycles of 30 s at 500 °C compared to a TRIDYN simulation of the SIMS depth profile (dashed lines) assuming the layers shown in the inset. Depth profiles are shown for ^28^Si (purple), Al (green), O (red), C (black), ^29^Si (solid dark blue and dashed light gray), and ^30^Si (solid light blue and dashed dark gray).

Figure 3 shows a ToF-SIMS depth profile of the implanted 150 nm Al film, post-layer exchange. The ^29^Si and ^30^Si abundances in the exchanged Si (at depths between 160 and 230 nm) were 0.2 and 0.1%, lower than the natural concentrations of 4.7 and 3.1% in the substrate (depths below 230 nm). C and O contamination was not detected in the exchanged Si layer but were seen in the exchanged Al, consistent with the EDX analysis.

Although the measured relative abundances of the Si isotopes could be trusted, note that matrix effects (where the secondary ion yield of an element depends on its surrounding matrix^32^) meant that ToF-SIMS could not be used to quantify relative elemental concentrations of Al, Si, C, and O. The ^28^Si signal was normalized to 100 in the substrate and the Al signal to 100 at the surface. The matrix effect precludes quantification of the concentration of Al in the exchanged Si. No information about nitrogen could be gained from the SIMS measurements. Nitrogen itself does not ionize well, and the signals from SiN^+^ and NH_4_^+^ cations were too weak to record during the ToF SIMS measurements.

A TRIDYN^33^ model of the SIMS sputter process predicted abrupt transitions at the Al/exchanged Si and exchanged Si/substrate interfaces. The poor agreement between experimental and TRIDYN profiles for the ^28^Si and Al at the ^28^Si/Al interface (∼125 nm in Figure 3) could be accounted for by the variation in surface Al and layer exchanged Si thickness over the large area sampled by SIMS (Figure 1B1). Good agreement between the abrupt experimental and TRIDYN profile shapes for the ^29^Si and ^30^Si at the substrate interface (∼210 nm in Figure 3) suggested little diffusion of Si from the substrate into the enriched film. The ToF-SIMS profile suggested Al diffusion into the bulk of the Si substrate beyond ∼250 nm, but we believe this tail to be a SIMS artifact. The horizontal depth scale reported by SIMS assumed a constant sputter rate. In reality, the sputter rate varies with elemental composition, which is modeled by TRIDYN. The 90 nm thickness of the enriched Si layer assumed by TRIDYN compares well with the average thickness observed over the TEM lamella (Figure 1B2). Accounting for variation of sputter rates between single- and polycrystalline material is outside the scope of TRIDYN. The Al reported in the enriched layer may be from pockets of trapped Al (such as those shown in the STEM-EDX analysis in Figure S2) rather than being homogeneously contained throughout the layer.

### Process Window

In a previous paper we used TRIDYN to model the implant energy and fluence and vacuum level process window of enrichment by direct implantation of ^28^Si^+^ into ^natural^Si.^21^ Here we have used TRIDYN to model the equivalent process window (over implant energy, fluence, and vacuum level) for Si implants into Al for ILE.

**Figure 4:**
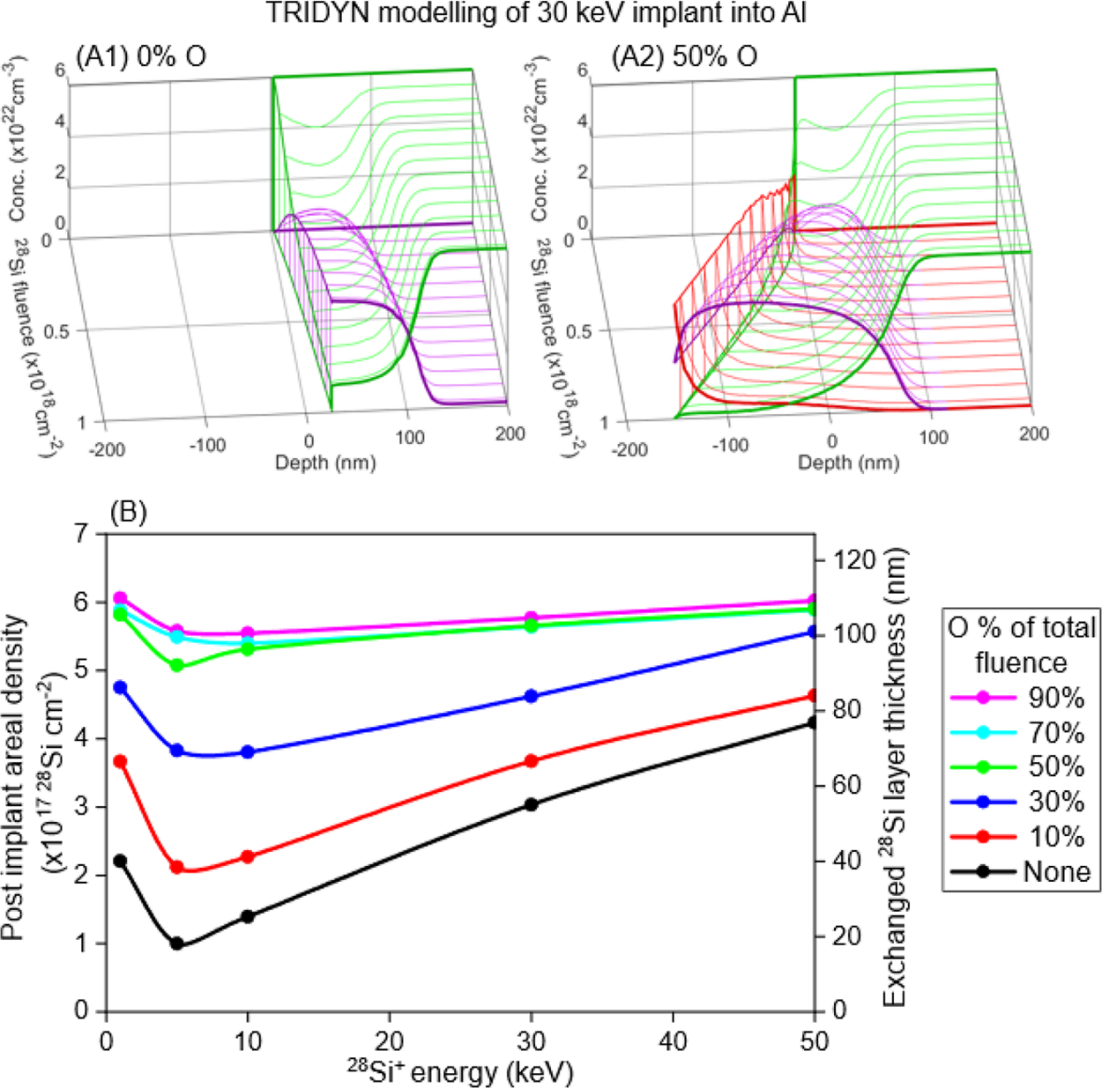
(A) TRIDYN models showing how the ^28^Si is implanted into the Al layer and either sputters away the Al target when O is not present (A1) or thickens the layer as the fluence increases for the case that the oxygen flux is the same as the ^28^Si ion flux (A2). (B) Areal density of ^28^Si atoms in the Al film after a ^28^Si^+^ fluence of 6.6 × 10^17^ cm^–2^ as a function of ion energy for varying O fluences (expressed as a percentage of the total fluence). The second vertical axis gives the thickness formed by the through layer exchange annealing (assuming each nm contains 5.5 × 10^15^^28^Si cm^–2^).

Figure 4A shows two TRIDYN models of the Al film surface evolution during ^28^Si 30 keV implants to a final fluence of 1 × 10^18^ cm^–2^ to illustrate the role of oxygen in the implant process. The model in Figure 4A2 includes zero energy O to represent oxygen containing molecules such as O_2_, H_2_O, and CO_2_ that arrive on the substrate surface from the residual vacuum and can then be recoil implanted into the layer. It should be noted that a total fluence of 50% O (and 50% ^28^Si) corresponds to the beam currents used and pressures present in the implanter during our experiments. The model predicts an exchanged layer thickness of 110 nm if the layer exchange step were 100% efficient which compares well to the 90 nm used in the TRIDYN SIMS model. A model with oxygen absent is shown in Figure 4A1 for comparison. Without O, the amount of ^28^Si saturates as the number of ^28^Si atoms incorporated reaches a concentration such that the rate of ^28^Si sputtering balances the flux of arriving ^28^Si. As ^28^Si ions also sputter away Al atoms, the film surface recedes throughout the implant. The presence of oxygen increases the average surface binding energies of the ^28^Si and Al atoms (see the Experimental Section for an explanation) which decreases both of their sputter yields. Hence, the total amount of ^28^Si accumulated in the layer can be seen to increase with the presence of O. In Figure 4A2 the layer incorporates ever more ^28^Si as the implant progresses. Oxygen is also incorporated into the layer, as shown experimentally in Figures 1 and 2, but this is not an issue for ILE as O is filtered out during subsequent layer exchange.

In direct implantation enrichment, the implanter vacuum level governs the O contamination in the ^28^Si layer. For ILE, the implanter vacuum can be used to incorporate a higher number ^28^Si atoms into the Al film for a given implant fluence. As the implanted oxygen has been shown to be gettered in the Al, the implanted O does not oxidize the epitaxial ^28^Si layer, unlike the case of direct ^28^Si implantation into Si.^21^Figure 4B summarizes the process results predicted by TRIDYN models over a range of energies and oxygen levels. In the absence of oxygen, the amount of ^28^Si deposited, and thereafter the thickness of the exchanged layer assuming all Si is incorporated into the epitaxial layer, would be reduced by self-sputtering. The trend of retained ^28^Si with energy can be attributed to the energy dependence of the sputtering. The maximum ^28^Si sputter rate is at ∼5 keV. As the amount of O is increased and the sputter rate reduced, the amount of incorporated ^28^Si increases up to 90% of the total ^28^Si fluence and becomes essentially independent of energy.

## Conclusions

### Process Capability

Our results demonstrate the use of a conventional industrial implanter can be combined with layer exchange to form extensive areas of epitaxial ^28^Si layers on a ^natural^Si substrate with isotopic enrichment of 99.7% ^28^Si. The use of thinner Al layers and higher anneal temperature improved upon our preliminary study that only produced discrete, nonepitaxial Si grains.

### Crystal Quality

The epitaxial layer was not completely continuous and contained regions where nucleation within the Al layer had competed with epitaxial growth. The epitaxial growth appears to be favored as it is observed above a layer of oxide or surface contamination in Figure 1B2, but any degradation of the epitaxial growth rate gives nucleation in the Al more of a chance to occur. Our study was limited by having to transfer wet oxide stripped wafers in atmosphere to Al deposition tools of uncontrolled cleanliness. Further studies outside the scope of this work^34^ where cluster equipment have enabled thorough oxide removal, surface cleaning, and Al deposition to be completed in vacuo have indicated that stringent removal of surface oxide and contamination from the Si substrate prior to Al deposition suppresses nucleation in the Al layer. For ILE to be successful, Si crystallization within the Al layer must be eliminated. It should be noted that the lack of any visible Si crystallization in the 100 nm thick film of this study (see TEM analysis in the Supporting Information, Section II) demonstrates that nucleation sites can be destroyed in the Al by implantation.

The speed at which ILE progresses during anneals (less than 30 s compared to hours for deposition based layer exchange^27^) and the formation of Si crystallites in the untouched Al before the anneal (Figure 1A3) suggest that pent-up potential energy in the implanted layer in addition to the Gibbs energy release was an important driver for the ILE process. The observation of the crystallites formed in the untouched Al before the anneal indicates that Si crystallization within the Al must be controlled at all stages of the ILE process.

### Isotopic Enrichment

The 3000 ppm enrichment achieved is within range of the 800 ppm of often-used quantum Si. The SIMS results suggested little self-diffusion from the ^natural^Si substrate (as expected at such low temperatures^20^), and so enrichment in this study appeared to be limited by the implanted minor isotopes that could be improved. The isotopic purity of an implant is principally limited by the mass resolution of the implanter (which can be compromised if mass resolving slits are widened to increase throughput or magnet drifts—especially during very long implants). This study was performed using an academic implanter in which the implant with μA level beam currents took 71 h to complete. An industrial implanter capable of producing 20 mA (and higher current) beams could implant a 300 mm wafer to 6.6 × 10^17^ Si cm^–2^ in 2.5 h. While we would expect such an implanter could improve the enrichment, further experiments would be required to measure the value achievable, confirm that throughput can maintained with the mass resolution required to ensure isotopic purity, and if there were any unanticipated second-order mechanisms that could limit enrichment.

### Aluminum Contamination

Compared to enrichment by implantation directly into the substrate, the nature of the layer exchange process has introduced the new problem of Al contamination of the Si. We proposed that the Al in the SIMS measurement of Figure 3 was dominated by trapped Al voids rather than Al within enriched Si, supported by the fact that STEM-EDX of Figure 2B could not detect Al in the exchanged layer. This trapped Al should be eliminated when Si crystallization within the untouched Al does not occur. However, it should be anticipated that Al will be present in the Si at its solid solubility level (0.75% at 500 °C),^27,35^ still too high for quantum grade Si which may be difficult to eliminate. Annealing strategies and gettering techniques (implant related or otherwise) such as those used to remove metallic contaminants from solar cells^36,37^ should be investigated.

### Other Elemental Contamination

Unlike direct implantation into Si, ILE is insensitive to surface oxidation because oxygen in the implanted region is not transferred to the exchanged layer. Indeed, surface oxidation was modeled to increase the retention of implanted ^28^Si by reducing the self-sputtering of the implant and largely eliminate ILE sensitivity to implant energy. A further advantage is that implanted isobaric contaminants (^14^N_2_, ^12^C^16^O) or recoil implanted surface contaminants were observed to be gettered within the implanted Al region and did not move into the enriched Si layer during layer exchange annealing. This enables the use of conventional positive ion beam implanters in which the isobaric beam contaminants cannot be mass filtered away.

### Layer Thickness Uniformity

Another new problem inherent to the ILE method is the variation in the enriched Si layer thicknesses observed to range between 90 and 180 nm for the of Si fluence used in this study. Although the average thickness meets the requirements for quantum devices, thickness uniformity will need to be improved. This is another issue that is likely to be mitigated by improved epitaxial growth uniformity promoted by better Si substrate cleaning and Al deposition.

More sensitive and absolute measurements are required to determine accurately the quality of the enriched layers. The sensitivity of STEM-EDX (∼1%) is not sufficient to accurately quantify the contamination levels of all elements in the layers. ToF-SIMS is more sensitive, but accuracy is spoiled by secondary ionization matrix effects, ion beam mixing, and the effects of differential sputtering rates through regions of different composition and crystallinity. These issues could be mitigated by taking SIMS measurements of higher quality enriched films after Al removal with comparison to specially prepared standard samples. The crystal defect density was estimated from cross-sectional TEM that only sample a small part of the substrate. A significant metrology would be to undertake spin lifetime measurements of Al atoms in the enriched layers. This could not only measure the concentration of Al present but would also be informative about sources of decoherence.^22^

### Process Window and Comparison to Direct Implantation

The experimental results and TRIDYN modeling suggest that ILE has a wide energy and vacuum level process window, allowing the use of conventional semiconductor foundry implanters.

The implant energy need only be chosen to be low enough such that for a chosen Al layer thickness, the Si substrate surface cannot be damaged. Beyond that constraint, the energy can be chosen to maximize implanter transmission and minimize sputtering. Direct implantation has limited energy windows (below 3 keV where beam transport is reduced) or above 45 keV (where high fluences are required to dilute the ^natural^Si) such that Si self-sputtering does not limit the isotopic enrichment possible.^21^

The ion beam fluences required for ILE are significantly (approximately order of magnitude) lower than those required for direct implantation into Si^22,21^ because mixing of the substrate’s natural Si into the implanted (or deposited) layer does not occur. Also, ^28^Si self-sputtering is reduced.

The isotopic enrichment is independent of fluence. The enriched layer thickness does not depend on the implantation energy but can be expected to scale with fluence of Si implanted into the Al. This study has produced layers of average 90 nm thickness, but some qubits do not require such a thick layer. For example, an acceptor-based qubit^2^ made using a 300 mm wafer CMOS process flow required a 20 nm thick layer enriched layer. An industrial implanter could complete the Si enrichment implant over 300 mm wafer for such a device in ∼30 min.

In summary, ILE has a compelling process window which potentially enables the conventional implanters to produce quantum grade enriched Si layers at reasonable throughput. However, the process results must first be improved. Crystallization in the Al layer must be eliminated to improve crystal quality, thickness uniformity, and Al content improved (which may be achieved by improving the quality of the substrate clean and Al deposition), and then the amount of Al must be further reduced (which may be achieved by the choice of annealing strategy and gettering techniques).

## Experimental Section

### Aluminum Deposition

The 25 × 25 mm^2^ Si coupons (cleaved from a 100 mm diameter Si wafer) were immersed in buffered hydrofluoric acid solution in a plastic Petri dish for ∼10 s to remove their native oxide before being coated with nominally 150 nm (and 100 and 200 nm) thick Al films using a Nordiko 2000 RF magnetron sputtering system. The actual film thicknesses were not measured.

### Ion Implantation

The coupons were implanted with ^28^Si/30 keV/6.6 × 10^17^ cm^–2^ using the Danfysik 1090 Implanter at the Surrey Ion Beam Centre. Residual vacuum levels of 10^–6^ mbar were present in the beamline and wafer end station during implantation. A ^28^Si energy of 30 keV was selected to be in a regime where beam transport through the implanter did not limit the highest beam current available (12 μA) and to be consistent with earlier studies.^21,30^ The implant took a total of 71 h to complete.

### Annealing

The implanted coupons were divided up into approximately 10 × 10 mm^2^ pieces for various annealing experiments at 500 °C, all performed under inert N_2_ atmospheres. The temperature selection reported in this paper was guided by conditions used for deposition-only Si–Al layer exchange^27^ using a thermocouple-controlled Jipelec JetFirst rapid thermal annealer. The sample was first heated and held 250 °C for 20 s to stabilize the thermocouple, before it was ramped up to 500 °C and held for 30 s. The cooldown cycle also paused at 250 °C. Several cycles were performed to see if significant process changes were evident in TEM cross-sectional images, but it appeared that layer exchange had fully completed within the first 30 s of the anneal.

### TEM Lamella Preparation

Two dual focused ion beams (FIB) with scanning electron microscope columns were used to prepare thin cross-section lamellae from bulk samples for analysis with a range of transmission electron microscopy (TEM) techniques. A TESCAN FERA3 Xe FIB was used to deposit a Pt strip to protect the sample surface and then sputter and lift out a 10 × 20 × 50 μm^3^ block which was then thinned to a thickness between 1 and 50 nm with a FEI Nova Nanolab 600 dual beam Ga FIB. Lamellae were fabricated parallel to the cleaved edges of samples to align the main {110} Si substrate crystal plane to the lamella surface. Scanning (S)TEM images of the lamellae could be taken using the Xe FIB.

### TEM Measurements

A lamella of the annealed sample was sent to EurofinsEAG for commercial TEM analysis. Images were collected using bright field (BF) and dark field (DF) STEM, TEM, and high-resolution (HR) TEM techniques using a FEI Tecnai TF-20 FEG/TEM operated at 200 kV, and energy dispersive X-ray spectroscopy (EDX) spectra were acquired using an Oxford INCA, Bruker Quantax EDS system. Crystal structure was measured by nanobeam diffraction. The TEM-based measurements of the as-implanted sample were performed using a new Thermo FEI Talos F200I TEM recently installed at the University of Surrey. Because of installation time, measurements were performed 7–10 months after the implants were carried out. EDX spectra were acquired using a Bruker X-Flash system. EDX was used to map the location of Si and Al in the samples displayed in this paper as false color images to indicate the predominant element.

### ToF-SIMS

Time-of-flight secondary ion mass spectrometry was used to depth profile the Si isotopes in the sample. An IONTOF TOF.SIMS 5 instrument employed a 25 kV Bi_3_^+^ ion source at 45° to the sample surface for surface spectroscopy over an area of 400 × 400 μm^2^ and a 3 kV Cs^+^ beam, also at 45°, to sputter away the sample for depth profiling.

## Computational Modeling

### TRIDYN Modeling

The Monte Carlo program TRIDYN^33^ was used to model ^28^Si implantation into Al and the SIMS sputter process. TRIDYN uses the binary collision approximation (BCA) to calculate implant profiles. Unlike static BCA codes such as TRIM/SRIM,^31^ TRIDYN accounts for dynamic target changes during implantation allowing high-fluence, multispecies implant profiles and sputter processes to be simulated. TRIDYN considers amorphous materials and does not account for channeling in crystalline materials. Standard built-in TRIDYN surface binding energies and atomic densities were used in all calculations using the standard approach of describing surface binding energies as a linear combination of binary interaction energies of the surface atoms scaled by their atomic fractions.^38^ Interaction energies were calculated from the heat of sublimation for Si–Si and Al–Al (4.7 and 3.36 eV, respectively). For O, only the interaction with Si or Al was considered as two interacting O atoms would make O_2_, which is gaseous. The Si–O and Al–O interaction energies (13.3 and 12.1 eV) were calculated from the heat of formation of SiO_2_ and Al_2_O_3_ compounds. The maximum O concentration in the substrate was limited to the stoichiometry of SiO_2_.

TRIDYN reported elemental depth profiles, elemental sputter yields, and surface growth or recession as the models progressed. Implants were modeled for Si ion energies of 1, 5, 10, 30, and 50 keV. In addition to the ion fluxes, zero energy oxygen atoms were included in the total particle fluxes of the models to simulate the arrival and sticking onto the substrate surface of oxygen containing molecules (O_2_, CO_2_, or H_2_O) present in the residual atmosphere in the implanter end-station. (In a TRIDYN model the calculation of a particle’s path is terminated once its energy falls below a cutoff energy of typically a few eV. Hence, zero energy particles immediately stop at the point of injection into the model, which is the surface of the substrate.) Models with oxygen fluxes of up to 90% of the total particle flux were considered, consistent with models used in our earlier studies.^21^ Oxygen fluxes of 50 and 90% of the total particle flux were calculated to relate to partial pressures of 1.2 × 10^–8^ and 1.1 × 10^–7^ mbar for the oxygen molecules, respectively (by calculating the molecular flux using the kinetic theory of gases for the partial pressures and considering the implant fluence and process times).

Ion beam mixing and sputtering during the ToF-SIMS analyses of the post-anneal samples were simulated using a simplified model of the post-layer exchange substrate consisting of a 150 nm thick layer of pure Al on top of a 90 nm thick ^28^Si Al layer containing 1% Al above a ^natural^Si substrate (Figure 3). This substrate was irradiated simultaneously with a high total particle flux comprising 90% Cs/3 keV and 10% Bi/8.3 keV (to account for the triatomic Bi ions) both at incident angles of 45°. A fluence of ∼6 × 10^17^ cm^–2^ ensured sufficient sputtering to simulate experimental results. The SIMS results were represented by plotting the sputter yields of each element vs fluence and converting the fluence values to sputter depth using the TRIDYN generated values of surface recession vs fluence. This model accounted for sputter effects such as broadening of interfaces by ion beam mixing and variation in sputter rate with changes in materials, but not channeling. It was beyond the scope of TRIDYN to predict the charge state of the sputtered atoms, and so matrix effects in secondary ion yields were not accounted for. To match the reported experimental data, the Al sputter yield was normalized to 100 at the surface (where the sample was pure Al). Likewise, the total Si sputter yield was normalized to 100 at the end of the model where the substrate was pure ^natural^Si. It should be emphasized again that this did not allow the amount of Al in the exchanged ^28^Si layer to be estimated because it did not account for the secondary ion yield matrix effect.

## Supporting Information Available

Isotopically enriched layers for quantum computers formed by ^28^Si implantation and layer exchange; analysis of circular features and epitaxial growth failure in 100 nm Al; additional top-down images, analysis through a circular defect showing it to be large polycrystalline Si, and description of 100 nm thick case in which layer exchange failed (PDF)

## Abbreviations

BCA: binary collision approximation
EDX: energy dispersive X-ray spectroscopy
ESR: electron spin resonance
ILE: implanted layer exchange
NMR: nuclear magnetic resonance
SEM: scanning electron microscopy
S/TEM: scanning/transmission electron microscopy
ToF-SIMS: time-of-flight secondary ion mass spectrometry.
