# Supplementary material for: Isotopically Enriched Layers for Quantum Computers Formed by 28Si Implantation and Layer Exchange
Source: ACS Appl Mater Interfaces. 2023 Apr 19;15(17):21609–17. doi: 10.1021/acsami.3c01112 (PMC10165600; doi:10.1021/acsami.3c01112)
Supplement: Supplementary file 1 — am3c01112_si_001.pdf [file am3c01112_si_001.pdf]

Supporting Information

Isotopically Enriched Layers for Quantum Computers Formed by ^{28}Si Implantation and Layer Exchange: Analysis of Circular Features and Epitaxial Growth Failure in 100 nm Al

Ella Schneider, Jonathan England

Surrey Ion Beam Centre, Advanced Technology Institute, University of Surrey, Guildford, GU2 7XH, United Kingdom

E-mail: e.b.schneider@surrey.ac.uk

List of supplementary information

I. TEM Analysis of a Lump Feature Formed during ILE in a 150 nm Al film

II. Layer exchange Failure with 100 nm Al Thick Film

III. References

I. TEM Analysis of a Lump Feature Formed during ILE in a 150 nm Al film:

Cross-sectional TEM analysis was performed across several of the circular lumps over a variety of anneal conditions and Al film thicknesses [1]. Figure S1 shows top down optical and SEM images of a 150 nm thick film implanted alongside the sample shown in the main paper but annealed for 1 hour in a Carbolite TZF 12/100 tube furnace under an inert N_2 atmosphere. The result is very similar to that shown in Figure 1(B1) of the main paper. The top-down optical microscope images display dark circular features of varying size, distributed evenly across a light background (Figures S1 (left)). In the complementary scanning electron microscopy (SEM) image (Figures S1 (right)), the features appear bright and shadows on the surrounding surface imply that they project out of the background layer.

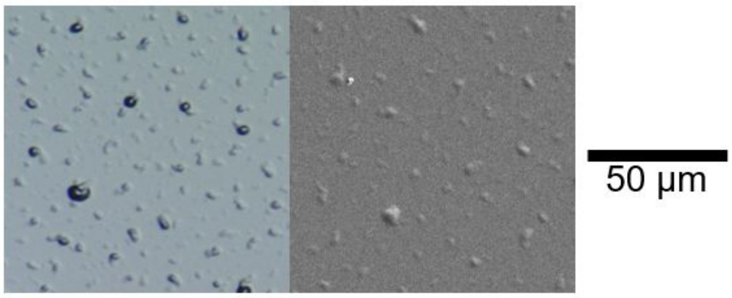

Figure S1. Left: Top-down optical microscopy of 150 nm Al film on Si implanted then annealed at 500˚C for for 1 hour in a Carbolite TZF 12/100 tube furnace under an inert N_2 atmosphere. Right: A complementary scanning electron microscopy (SEM) image taken using an FEI Quanta ESEM.

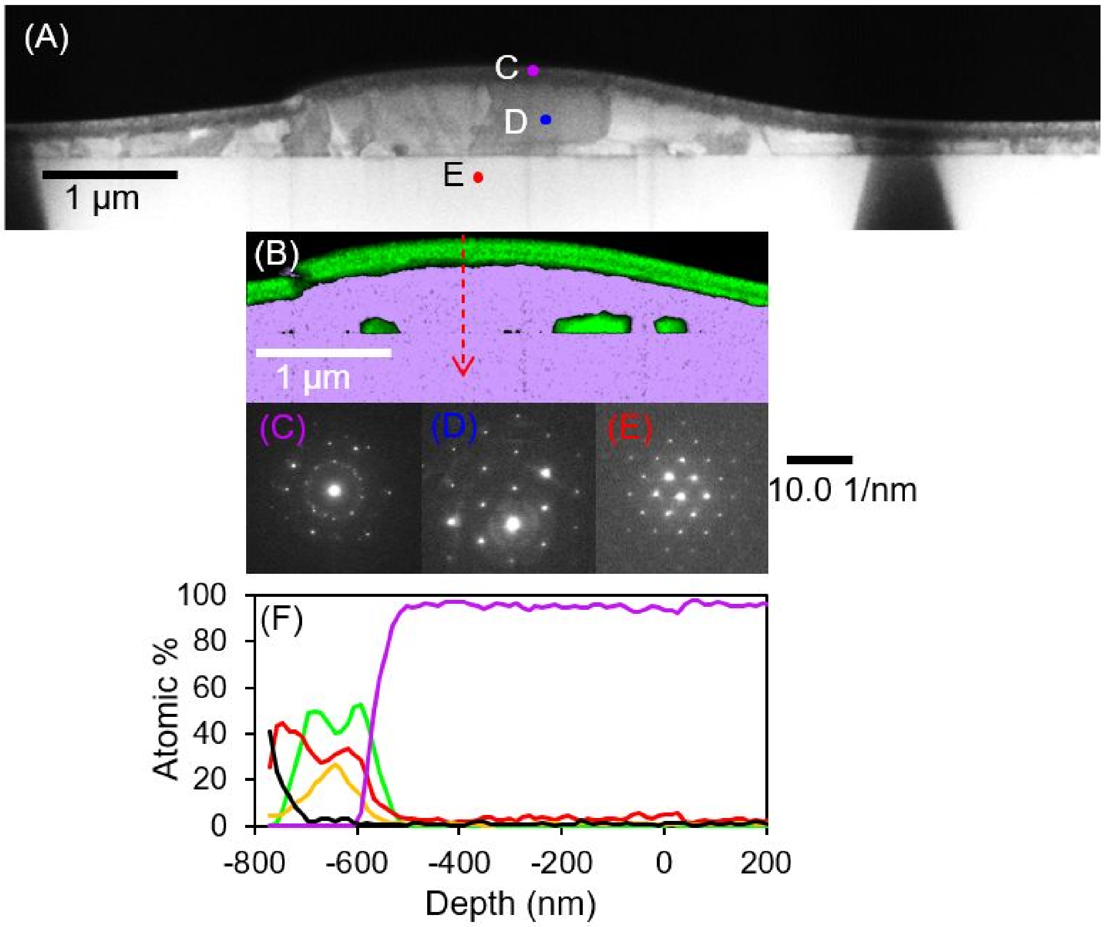

Figure S2. (A) STEM image of cross-section taken through a dark circular feature from the sample shown in Figure S1. (B) False-colour STEM-EDX map of Si (purple) and Al (green)

X-rays. (C-E) nanobeam diffraction patterns taken in (C) the surface Al layer, (D) a Si grain and (E) the Si substrate. (F) Semi-quantitative STEM-EDX depth profile taken across red dashed arrow shown in (B) Elements shown: Si (purple), Al (green), O (red), C (black) and N (orange).

A TEM lamella made across one of these dark circular features imaged in Figure S2(A) confirms that the features did indeed project out of the background. A STEM-EDX map of Figure S2(B) shows the large lump to consist of predominantly Si located beneath the tri-layer that indicates the implanted region (see discussion later in this section). The interface at the original Si substrate surface is sharp across the whole image which implies little intermixing of the substrate and exchanged Si during layer exchange. The well-defined diffraction pattern of one of the Si grains (Figure S2(D)) implies the formation of a large crystal at a different orientation to the substrate. The STEM-EDX map (Figure S2(B)) reveals the presence of Al inclusions at the substrate interface which appear to have been trapped by lateral over-growth of two large (~600 nm thick) Si crystals from either side. This evidence implies heterogeneous nucleation of Si followed by growth of these nuclei to the exclusion of epitaxial growth on the substrate. The STEM-EDX line profile (Figure S2(F)) shows little contamination present in the

enriched Si region. O, N and C contamination was gettered to the dark contrast region of the surface Al. The dark region (which is polycrystalline according to diffraction pattern of Figure S2(C)) creates the appearance of a tri-layer at the sample surface. We attribute this region to decoration of implant damage during the anneal.

Not all of the features formed during layer exchange were Si rich. Analysis of a feature formed during a 5 minute 500°C layer exchange anneal of an implanted 200 nm thick Al film consisted of polycrystalline Al grains [1]. This means the crystallization and lateral movement of the Al as well as the Si needs to be considered during layer exchange.

II. Layer exchange Failure with 100 nm Al Thick Film:

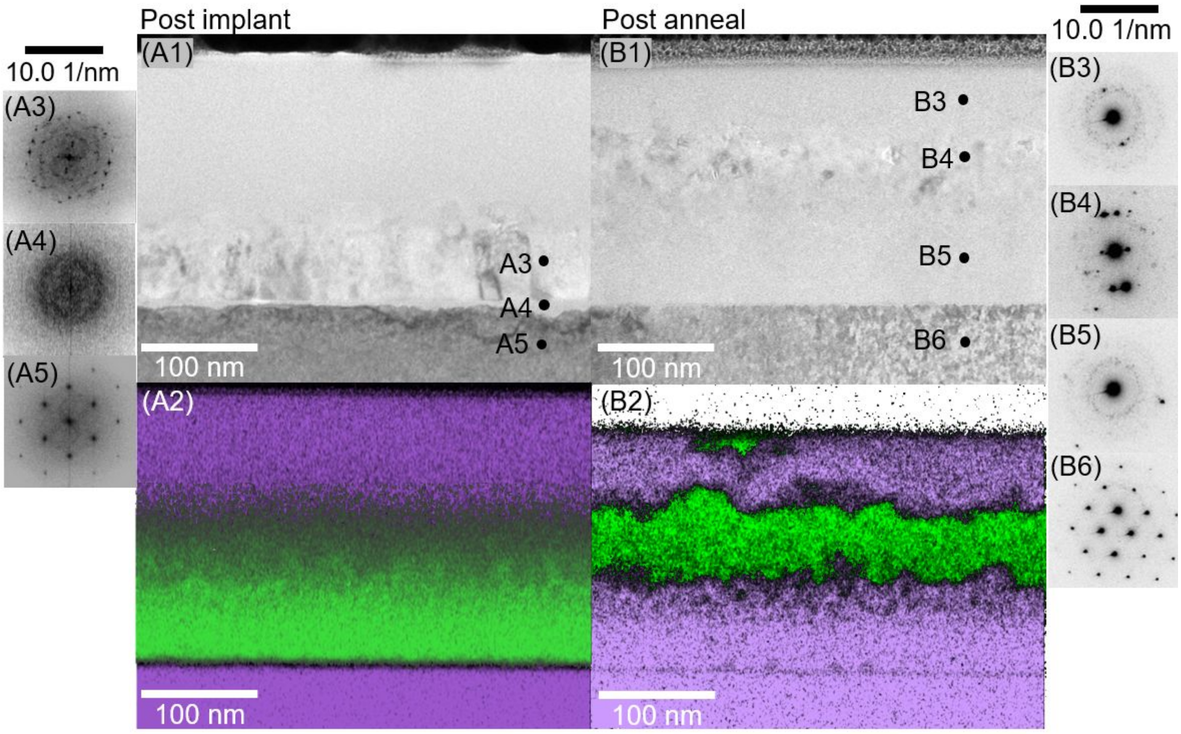

Figure S3. TEM analysis of a 100 nm thick Al film on Si, implanted with ^{28}Si/30 keV/6.6x10^{17}cm^{-2} and annealed for 1 hour at 500°C in a Carbolite TZF 12/100 tube furnace. (A1-B1) BF-TEM images, (A2-B2) STEM-EDX false color maps of Si (purple) and Al (green) X-rays. (A3-A5) high resolution TEM fast Fourier transform patterns and (B3-B6) nanobeam diffraction patterns taken in locations indicated in (A1-B1).

Successful layer exchange and epitaxial growth proceeded in the 150 nm Al layers and a thicker 200 nm film (not shown) [1] but failed in a 100 nm thick Al layer. High resolution TEM fast Fourier transform patterns in Figure S3 showed that the Al / Si substrate interface was

damaged by the implant into the 100 nm Al film. Figure S3(A4) showed that the Al / Si substrate interface was amorphized. The variation in amorphization thickness (10-30 nm) could be attributed to varying ranges of the implanted Si ions that depended on the crystal orientation of the Al grains; grains with a crystallographic direction aligned with the beam direction allowed deeper penetration of the Si ions than grains that were randomly oriented to the ion beam. The region of the 100 nm Al layer close to the Si substrate was exposed to a relatively low fluence compared to the top of the Al layer and had retained its crystallinity; it is known that metals are harder to amorphize than Si [2].

Significantly, this layer did not undergo layer exchange. The BF-TEM images, STEM-EDX maps and electron diffraction patterns in Figure S3(B1), S3(B2) and S3(B3-B6) showed that Si was expelled from the center of the Al layer to form amorphous Si rich layers above the interface and at the surface. The top and bottom layers, that would have been Si free after successful layer exchange, were seen to consist predominantly of amorphous Si containing some small poly-crystals. The lack of Si crystallization suggests that the implant had destroyed all nucleation sites throughout the Al layer and halted epitaxial growth onto the substrate. Homogeneous nucleation was not observed to occur within the one hour of the anneal. The

absence of removal of Si via epitaxial growth has not eliminated Si from the surface of the Al film.

The BF-TEM image, STEM-EDX map and nano-beam diffraction patterns in Figure S3(B1), S3(B2), S3(B3-B6) showed that the middle of the layer was predominantly composed of polycrystalline Al. Figure S4 showed that location of this Al layer appears where O, N and C are gettered during successful layer exchange of the 150 nm sample.

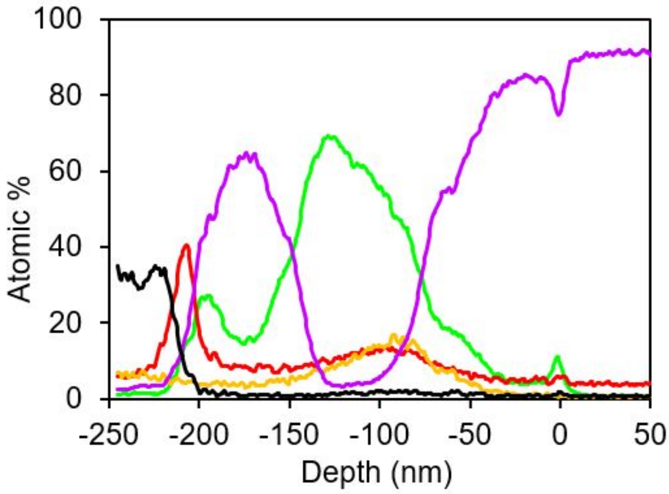

Figure S4. Semi-quantitative STEM-EDX depth profile of a 100 nm thick Al film on Si, implanted with ^{28}Si/30 keV/6.6x10^{17}cm^{-2} and annealed for 1 hour at 500°C in a tube furnace. Elements shown: Si (purple), Al (green), O (red), C (black) and N (orange).

VI. References:

[1] Schneider, E. B. Fabrication of Isotopically Pure ^{28}Si and ^{74}Ge Layers for Quantum Computers using Ion Implantation and Metal Induced Layer Exchange, PhD Thesis, University of Surrey, Guildford, 2023.

[2] Ziemann, P. Amorphization of Metallic Systems by Ion Beams Materials Science and Engineering 1985, 69, 1, 95-103.
